# Removing Foreign Bodies from the Æsophagus

**Published:** 1877-11

**Authors:** 


### REMOVING FOREIGN BODIES FROM THE ŒSOPHAGUS.

Dr. Reeves says, in the Melbourne *Medical Record*, "The plan is exceedingly simple, and it may be said to be the same as that adopted for extracting pieces of cork from bottles; the only difference being that the ends of the wire are bent outward and upward, instead of inward, and in place of a wire ring to compress the prongs, a large-sized catheter is passed down to their extremities, which, when the wires are passed beyond the obstruction, is drawn up to allow the prongs to expand. The instrument is made in the following manner: Three or four pieces of elastic wire, from twenty to twenty-four inches long, are twisted together down to within half an inch of their extremities, the extreme ends of which are curved outward and upward and carefully filed down, to prevent their injuring the coats of the canal. A piece of large-sized catheter of the same length is passed down, to prevent the prongs expanding, the extremities fitting well over the ends of the catheter, to admit of the instrument readily passing beyond the obstruction, without driving it deeper into the canal. The instrument, with the prongs closed, is passed down the gullet, well beyond the piece of bone or money, and the tube is then drawn up, the prongs expanding, bringing with the foreign body. Some difficulty may be experienced with fine long fish bones, from the extremities of the prongs not being able to obtain sufficient hold upon them to loosen them from their attachment to the mucous membrane; but if a little floss silk is passed loosely from the extremity of one prong to the other, this will be overcome."

